# Diagnostic Performance of Guideline-Recommended B-Type Natriuretic Peptide Thresholds for Detecting Elevated Left Ventricular End-Diastolic Pressure

**DOI:** 10.3390/diagnostics16162629

**Published:** 2026-08-19

**Authors:** Weiwei Jin, Wei-min Brian Chiu, Asia Krupa, Christopher Jerdonek, Kendra R. Cook, Derek Rinderknecht

**Affiliations:** 1 Ventric Health, 117 E Colorado Blvd., Suite 510, Pasadena, CA 91105, USA; brian@ventrichealth.com (W.-m.B.C.); asiakrupa@ventrichealth.com (A.K.); chris@ventrichealth.com (C.J.); kendra@ventrichealth.com (K.R.C.); 2 School of Biomedical Engineering & Imaging Science, King’s College London, London WC2R 2LS, UK

**Keywords:** B-type natriuretic peptide, left ventricular end-diastolic pressure, heart failure, hemodynamics, cardiac biomarker

## Abstract

**Background:** B-type natriuretic peptide (BNP) is widely used in the diagnosis, risk stratification, and management of heart failure (HF). However, its diagnostic performance for detecting elevated left ventricular filling pressure, particularly at lower thresholds, remains uncertain. Left ventricular end-diastolic pressure (LVEDP), measured during cardiac catheterization, provides direct evidence of abnormal left ventricular filling pressure and is a well-established indicator for HF. This study evaluated the diagnostic performance of guideline-recommended BNP thresholds for detecting elevated LVEDP. **Methods:** Data were analyzed from 154 patients referred for left heart catheterization (mean age, 66 ± 10 years; 64% male). LVEDP was measured directly using Millar Mikro-Tip pressure catheters. BNP was measured during the same visit. At a BNP threshold of 35 pg/mL, sensitivities for detecting LVEDP >15, >20, and >25 mmHg were 74%, 88%, and 92%, respectively, with corresponding specificities of 48%, 47%, and 42%. **Results:** At a BNP threshold of 100 pg/mL, sensitivities were 43%, 63%, and 75%, respectively, with corresponding specificities of 74%, 75%, and 71%. The corresponding areas under the receiver operating characteristic curve were 0.65, 0.75, and 0.81 for LVEDP >15, >20, and >25 mmHg, respectively. **Conclusions:** BNP demonstrated greater diagnostic accuracy at higher LVEDP thresholds but had limited sensitivity at lower thresholds, which may reduce its ability to detect hemodynamic abnormalities relevant to the evaluation of Stage B HF. BNP should therefore be interpreted in the context of a broader clinical assessment and additional diagnostic findings, particularly when the goal is to identify patients with less pronounced elevations in filling pressure.

## 1. Introduction

Early identification of abnormal filling pressure is important for heart failure (HF) risk stratification and clinical management. Elevated left ventricular filling pressure is a fundamental hemodynamic abnormality in HF that contributes to congestion and disease progression [1]. In the current Universal Definition of HF, elevated filling pressure, along with increased natriuretic peptide concentrations, may provide objective evidence of HF [2].

Elevated left ventricular end-diastolic pressure (LVEDP) can result from impaired ventricular relaxation, reduced ventricular compliance, systolic dysfunction, abnormal hemodynamic loading, or a combination of these factors [2,3]. As an objective hemodynamic indicator of left ventricular (LV) dysfunction, elevated LVEDP is relevant across the spectrum of HF phenotypes. This includes HF with preserved ejection fraction (HFpEF) [4], where filling pressures may be elevated despite the absence of clear structural findings.

B-type natriuretic peptide (BNP) is a widely used blood-based biomarker for the diagnosis, risk stratification, and monitoring of HF [5,6]. BNP is secreted by ventricular cardiomyocytes in response to increased wall stress and volume overload. BNP concentrations are therefore associated with cardiac filling pressures and the severity of hemodynamic congestion [5,7]. Current European and American HF guidelines recommend natriuretic peptide testing as an initial diagnostic tool in patients with suspected HF, particularly in outpatient and primary care settings where access to echocardiography or invasive hemodynamic assessment may be limited [2,3]. In these settings, BNP is particularly valuable for its high negative predictive value. This makes it an effective rule-out test for HF when levels are below established diagnostic thresholds.

Despite its widespread clinical use, the diagnostic value of BNP for identifying patients with elevated LVEDP remains incompletely characterized [8]. Numerous studies have examined the relationship between BNP and echocardiography-based estimations of left ventricular filling pressure [9,10,11], whereas fewer studies have investigated the relationship between BNP and directly measured left-sided filling pressures [12,13,14,15]. In a study of 65 patients with chronic LV dysfunction referred for right heart catheterization, an area under the receiver operator curve (AUC) of 0.74 was reported for a BNP threshold of 100 pg/mL to identify pulmonary capillary wedge pressure (PCWP) > 15 mmHg [12]. A separate investigation of 72 patients with symptomatic LV dysfunction undergoing right and left heart catheterization found that BNP correlated strongly with both LVEDP (r = 0.79, *p* < 0.001) and PCWP (r = 0.71, *p* < 0.001) [13]. In another study a more modest correlation between BNP and LVEDP was observed in 33 clinically stable outpatients with chronic HF (r = 0.50, *p* < 0.02) [14]. Among patients with preserved LV systolic function undergoing cardiac catheterization, an optimal BNP threshold of 33 pg/mL yielded an AUC of 0.74, with 72% sensitivity and 70% specificity for identifying LVEDP > 16 mmHg [15]. These studies support the diagnostic utility of BNP for identifying elevated filling pressures, but they provide limited information regarding the diagnostic performance of guideline-recommended BNP thresholds across different LVEDP levels.

This study evaluates the diagnostic performance of guideline-recommended BNP thresholds for identifying elevated LVEDP at different levels, using high-fidelity invasive LV pressure measurements in patients undergoing nonemergent left heart catheterization.

## 2. Methods

**Figure 1:**
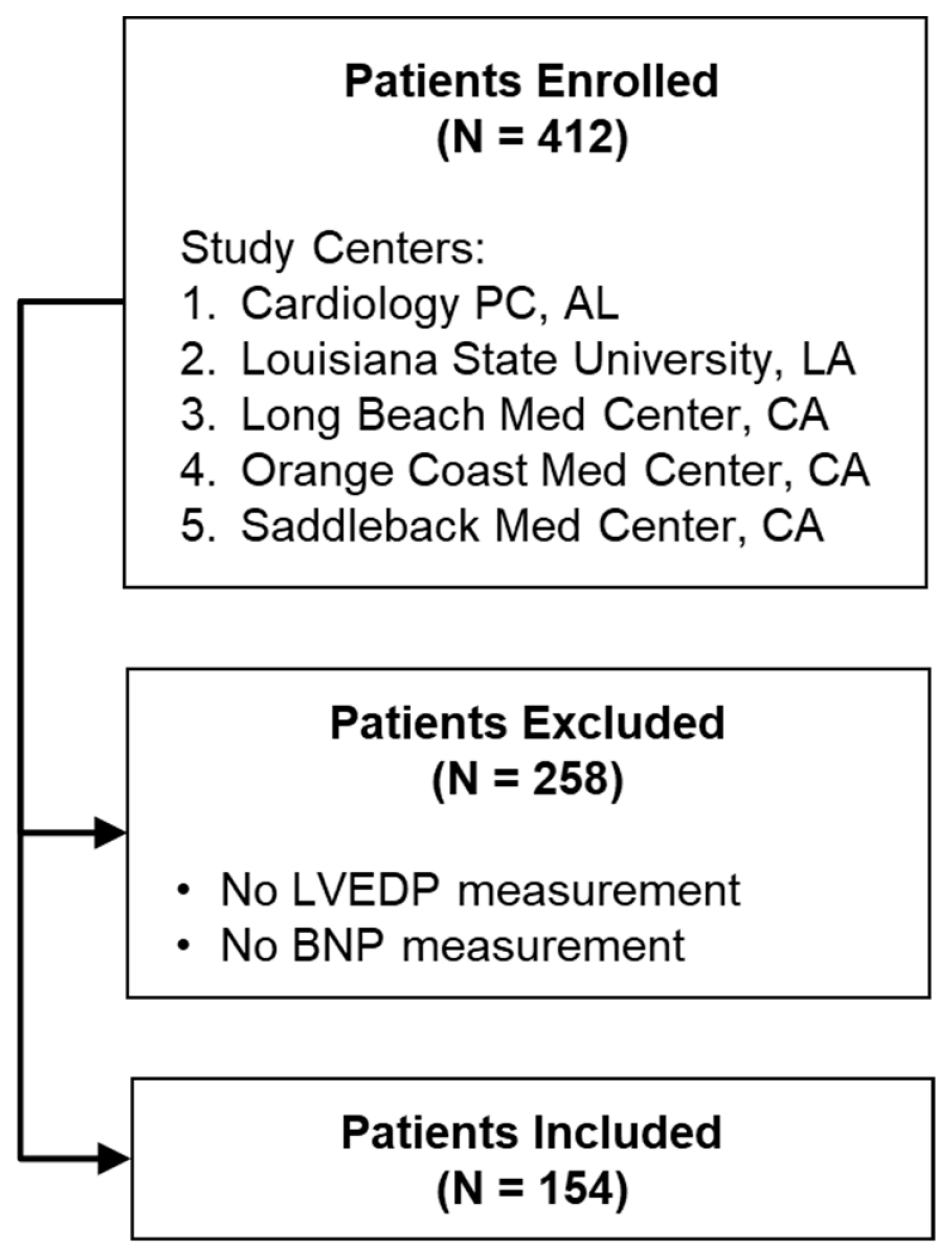
Patient enrollment flowchart.

This study is a secondary analysis of the cohort from the “Assessment of the Vivio System for the Non-Invasive Estimation of Left Ventricular Diastolic Pressure” (NCT05633004). The data in this manuscript comprises 412 patients from five study centers across the United States who underwent non-emergent left heart catheterization between September 2021 and October 2022. The catheterizations used femoral or radial access, per clinical practice. Patients with either acute coronary syndrome or myocardial infarction within a week of the procedure, cardiogenic shock, or the need for inotropic or mechanical circulatory support were excluded from the enrollment. A total of 258 patients were excluded from the 412 due to missing BNP measurements or poor-quality catheterization data. Because BNP collection was optional in the original study protocol, 235 (92%) of the 258 patients were excluded due to unavailable BNP values. Comparisons between patients with and without BNP measurements showed no significant differences in age (*p* = 0.057), sex (*p* = 0.664), or HF status (*p* = 0.954). The final analysis included 154 patients. Figure 1 shows a flow diagram of inclusions and exclusions.

All participants provided written informed consent. The study protocol was approved by the following institutional review committees: Advarra; the MetroWest Medical Center Institutional Review Board; and the Institutional Review Board at Louisiana State University Health Shreveport. Due to the sensitive and protected nature of the data collected for this study, the data are not publicly available and will remain under embargo for five years. Thereafter, deidentified data may be made available upon reasonable request to the corresponding author, subject to IRB approval and applicable data use agreements.

Blood samples for BNP measurements were drawn immediately before LVEDP assessments, either in the pre-procedure area or via the catheterization sheath. LVEDP was measured using Millar Mikro-Cath pressure catheters(Millar, Houston, TX, USA), with catheter and electrocardiogram (ECG) tracings recorded via a PowerLab DAQ system (www.adinstruments.com). LVEDP values were computed using the left ventricular pressure corresponding to the peak of the R-wave on the ECG [16]. Patient characteristics and disease conditions were obtained from electronic health records and documented medical history.

Receiver operating characteristic (ROC) curves were used to assess BNP’s ability to detect elevated LVEDP. BNP’s diagnostic performance was evaluated at the BNP thresholds from the 2021 ESC HF guidelines [3] for non-acute and acute HF, namely 35 pg/mL and 100 pg/mL, respectively, with sensitivity and specificity analyzed at LVEDP thresholds of 15, 20, and 25 mmHg.

All statistical analyses and diagnostic performance evaluations were performed using Python code, specifically Python 3.10. The SciPy 1.16.3 and statsmodels 0.14.5 Python libraries were used for statistical analyses, and scikit-learn 1.7.2 was used for the calculation of diagnostic performance metrics. In the statistical analyses, the continuous variables, including age and body mass index (BMI), were summarized as mean ± standard deviation (SD) and compared between groups using two-sample *t*-tests. The categorical variables, including sex and the binary classification of elevated versus non-elevated LVEDP, were summarized as counts and percentages and compared using chi-square tests.

## 3. Results

Patient characteristics and disease conditions for the 154 patients included in the analysis are summarized in Table 1 (stratified by various LVEDP subgroups) and Table 2 (stratified by various BNP subgroups). The cohort had a mean age of 66 ± 10 years, with 64% male, 80% diagnosed with hypertension, and over one third with diabetes. The tables also present summary statistics for BMI, HF diagnoses, and comorbidities including chronic kidney disease, atrial fibrillation, and valvular heart disease across the groups categorized by the LVEDP and BNP thresholds. As shown in Table 1, BNP levels were significantly higher among patients with elevated LVEDP at all three thresholds (>15, >20, and >25 mmHg), and BMI was also significantly higher at each LVEDP threshold. Additionally, as shown in Table 2, patients with elevated BNP differed significantly from those with lower BNP levels with respect to age and the prevalence of diabetes, chronic kidney disease, atrial fibrillation, valvular heart disease, and prior HF. LVEDP values also were significantly higher among patients above both BNP thresholds (35 and 100 pg/mL) compared to those below. Multivariable analysis (Table S1) yielded similar findings, identifying chronic kidney disease and prior HF as strong predictors of elevated BNP levels.

**Figure 2:**
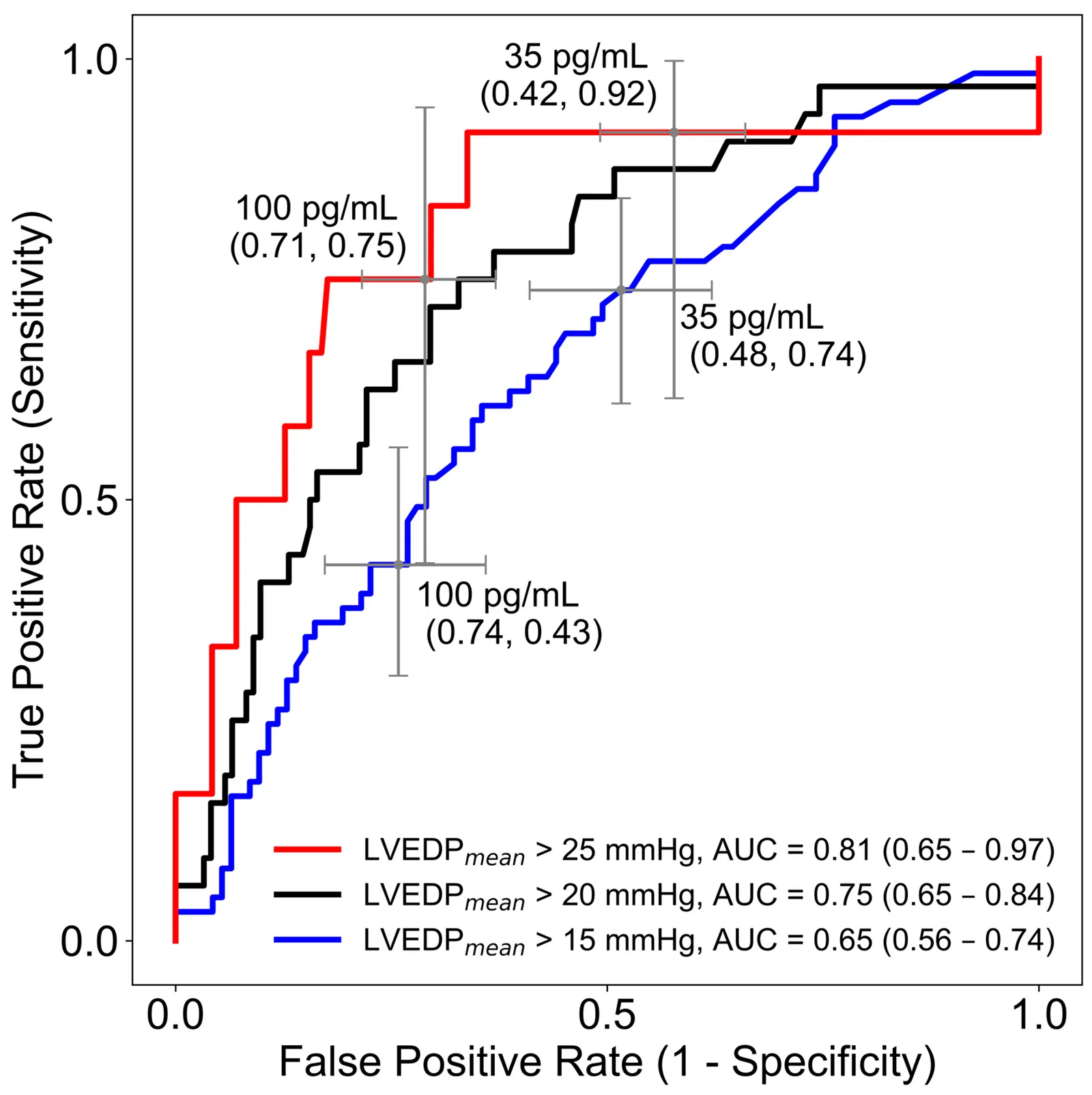
Receiver operating characteristic (ROC) curves corresponding to LVEDP thresholds of 15 mmHg (blue), 20 mmHg (black), and 25 mmHg (red), with area under the ROC curve (AUC) values and the corresponding 95% confidence intervals.

The diagnostic utility of BNP for identifying elevated LVEDP was assessed using ROC curve analysis. Figure 2 displays ROC curves for LVEDP thresholds of 15 mmHg (blue), 20 mmHg (black), and 25 mmHg (red). On the 15 mmHg and 25 mmHg ROC curves, the BNP thresholds for acute HF (100 pg/mL) and non-acute HF (35 pg/mL) are marked, along with their respective specificity and sensitivity values and 95% confidence intervals. Diagnostic performance varied according to both the BNP decision threshold and the extent of LVEDP elevation. At the 35 pg/mL BNP threshold, sensitivity increased from 74% for LVEDP > 15 mmHg to 88% for LVEDP > 20 mmHg to 92% for LVEDP > 25 mmHg, while specificity remained low at 48%, 47%, and 42%, respectively. At the 100 pg/mL BNP threshold, sensitivity was 43%, 63%, and 75% for LVEDP >15, >20, and >25 mmHg, respectively, with corresponding specificities of 74%, 75%, and 71%. Furthermore, for LVEDP > 15 mmHg, positive predictive values remained below 60%, and the negative predictive values remained below 80%, whereas for LVEDP > 25 mmHg, negative predictive values exceeded 95% at both BNP thresholds. ROC analysis also demonstrated improved discrimination at higher LVEDP thresholds, with AUC of 0.65, 0.75, and 0.81 for LVEDP > 15, >20, and >25 mmHg, respectively.

**Figure 3:**
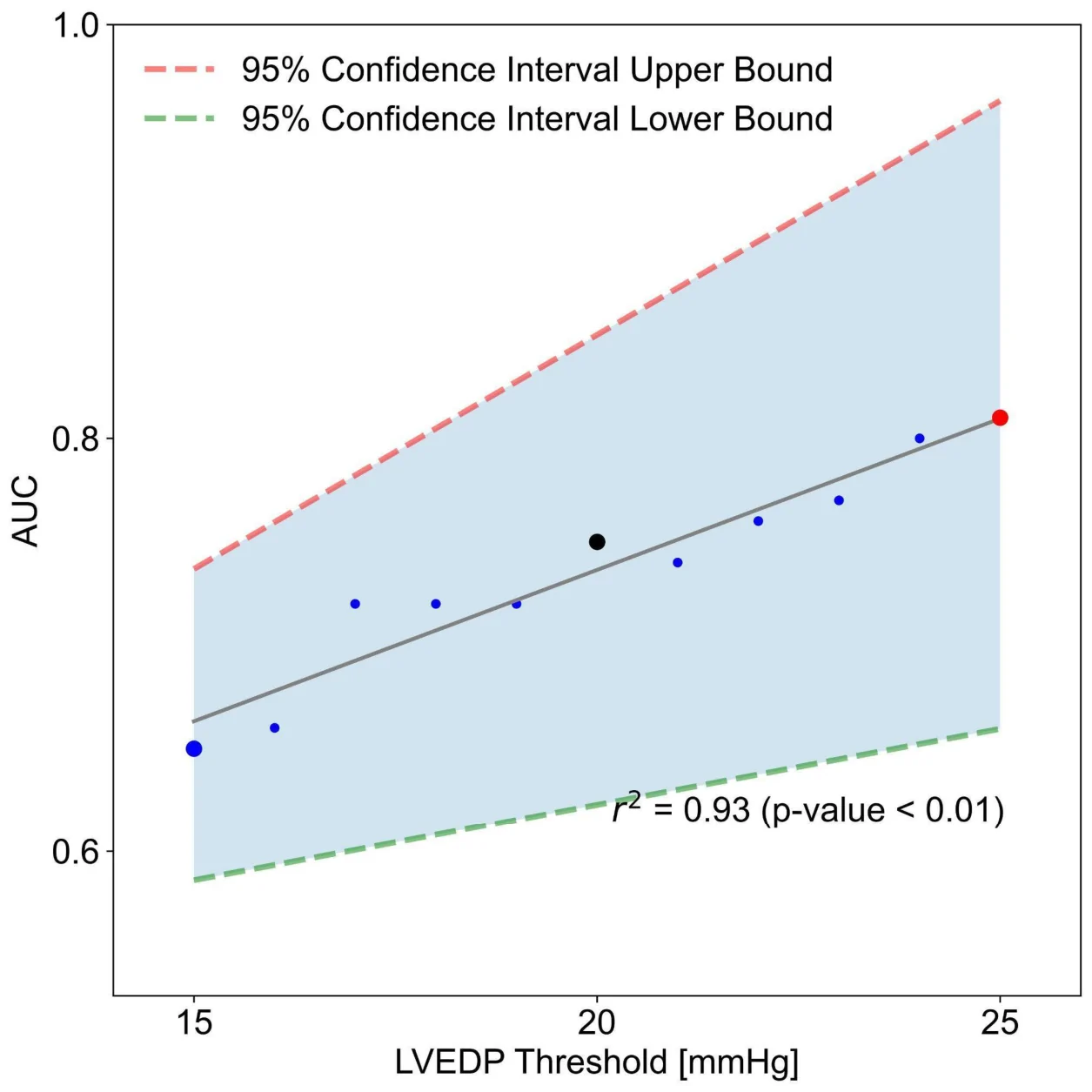
Area under the receiver operating characteristic curve (AUC) as a function of LVEDP thresholds. Blue, black, and red dots indicate the AUC at LVEDP thresholds of 15, 20, and 25 mmHg, respectively.

To further characterize this relationship, AUC was also calculated in 1 mmHg increments across the same 15–25 mmHg range used in the ROC curve analysis. As shown in Figure 3, a strong linear relationship was observed between AUC and increasing LVEDP thresholds (R^2^ = 0.93, *p* < 0.01), showing that BNP’s ability to discriminate improved progressively as LVEDP increased.

The complete diagnostic performance metrics, including positive and negative predictive values and likelihood ratios with their corresponding 95% confidence intervals, are presented in Table 3. Corresponding results from the sensitivity analysis excluding patients with chronic kidney disease and prior HF are shown in Tables S2 and S3, respectively.

Sensitivity analyses demonstrated findings consistent with the primary analysis. Diagnostic performance was relatively poor for identifying LVEDP > 15 mmHg but improved at the LVEDP > 20 mmHg threshold. At the LVEDP > 25 mmHg threshold, however, estimates became less precise because of the limited number of patients in the subgroups, particularly after excluding patients with a prior HF diagnosis, which left only 4 patients above this threshold.

## 4. Discussion

BNP is released in response to ventricular wall stress and elevated preload, making it a valuable biomarker for detecting changes in ventricular pressure and congestion [17,18]. In practice, BNP is a clinically reliable biomarker for ruling out HF, particularly as disease severity increases [19,20,21] and therefore is widely used in the diagnosis and monitoring of HF. In this prospective cohort of patients undergoing left heart catheterization, BNP demonstrated limited diagnostic performance for identifying elevated LVEDP at lower thresholds.

Diagnostic performance was influenced by both the selected BNP decision threshold and the degree of LVEDP elevation (Table 3). The diagnostic accuracy of BNP improved as the LVEDP threshold increased. Use of the lower BNP threshold for non-acute HF improved sensitivity at the expense of specificity, resulting in higher negative predictive values across all LVEDP thresholds. In general, BNP exhibited similar diagnostic performance to prior invasive studies only at higher LVEDP thresholds [22]. For example, Parsonage et al. and Jaubert et al. each reported an AUC of 0.74 for detecting elevated filling pressures at thresholds of PCWP > 15 mmHg and LVEDP > 16 mmHg, respectively, comparable to the AUC of 0.75 observed in the present cohort at LVEDP > 20 mmHg. These results are consistent with previous studies demonstrating that BNP is more robust for ruling out HF than for confirming its presence [23,24].

Iwanaga et al. demonstrated that BNP more closely reflects left ventricular end-diastolic wall stress than LVEDP itself [25], providing a possible physiological explanation for the false-negative BNP results observed in the present study. Compensatory ventricular remodeling may partially offset increases in wall stress, attenuating BNP release even when filling pressures are elevated. This mechanism may also explain the improved diagnostic discrimination observed at higher LVEDP thresholds, where wall stress rises more consistently with filling pressure.

Several established non-cardiac factors are also known to influence circulating BNP levels and act as confounders, potentially compromising diagnostic accuracy [26,27]. Consistent with previous studies, elevated BNP concentrations in this cohort were associated with older age and several comorbid conditions, including diabetes, chronic kidney disease, atrial fibrillation, and prior HF (Table 2). Multivariable analysis (Table S1) identified chronic kidney disease and prior HF as the strongest independent correlates of elevated BNP, suggesting that BNP reflects not only cardiac filling pressures but also the cumulative burden of cardiovascular and systemic disease.

Sensitivity analyses excluding patients with chronic kidney disease (Table S2), as well as those with a prior history of HF (Table S3), did not substantially change the overall interpretation of BNP’s performance. Specificity improved in these analyses, particularly after excluding patients with a prior HF diagnosis. However, these gains came at the expense of sensitivity. This trade-off suggests that certain confounding comorbidities can contribute to BNP elevation but do not fully explain its limited sensitivity for detecting elevated filling pressures.

In contrast, age and other comorbidities were not significantly associated with elevated LVEDP in this cohort (Table 1). A prior diagnosis of HF was significantly associated with elevated LVEDP only at thresholds above LVEDP > 20 mmHg. Patients with elevated LVEDP also had a higher BMI, whereas BMI did not differ significantly between patients with and without elevated BNP (Table 2). Previous studies have reported reduced BNP concentrations in individuals with obesity, potentially due to altered natriuretic peptide metabolism or secretion [28,29]. However, the lack of a significant BMI difference between BNP groups in this study suggests that obesity is unlikely to fully explain the false-negative BNP results observed here, although this finding may also reflect a limited range of BMI values in this cohort.

Beyond these established confounders, the limited sensitivity of BNP at LVEDP thresholds has important implications for the early detection of HF. In the STOP-HF trial [30], BNP was evaluated as a screening tool to identify asymptomatic individuals with cardiovascular risk factors for further evaluation through echocardiography and collaborative cardiovascular care, resulting in a reduction in the prevalence of asymptomatic left ventricular dysfunction with or without newly diagnosed HF. This phenotype is now recognized in the American Heart Association guidelines as Stage B HF [2]. Importantly, however, the success of STOP-HF reflected a multimodal screening and management strategy rather than the diagnostic performance of BNP alone. The substantial number of false-negative BNP results observed at lower LVEDP thresholds in the current study suggests that BNP-based screening may fail to detect elevated filling pressures, a hemodynamic abnormality relevant to the evaluation of Stage B HF, despite BNP concentrations below guideline-recommended thresholds.

These findings may be particularly relevant in HFpEF. Current HF guidelines recognize that natriuretic peptide concentrations may remain below diagnostic thresholds in some patients with HFpEF, particularly early in the disease course and in individuals with obesity [3,31]. The present work provides invasive hemodynamic support for this limitation by demonstrating that BNP may remain below guideline-recommended thresholds despite objectively elevated LV filling pressures. These results support the use of BNP as one component of a comprehensive diagnostic evaluation rather than as a standalone indicator of elevated filling pressures, particularly in patients undergoing evaluation for possible Stage B HF or early HFpEF.

## 5. Limitations

Several limitations should be considered when interpreting these findings. Firstly, this study evaluated BNP rather than NT-pro BNP. NT-pro BNP may be more advantageous, as it has been reported to be more sensitive to changes in cardiac filling pressures and is generally associated with greater assay standardization across analytical platforms. Secondly, BNP measurements were not standardized across study centers. Differences in sample collection methods, assay platforms, and laboratory protocols may have introduced measurement bias and reduced the comparability of BNP values across patients, potentially affecting the observed associations between BNP and LVEDP. However, this reflects routine clinical practice, where BNP testing is frequently performed using different assays and laboratory protocols across clinical practices. In addition, referral criteria, catheterization conditions, and sedation practices were not standardized across clinical sites. Variations in patient selection and procedural factors may have influenced LVEDP measurements and introduced additional inter-site variability, thereby affecting the comparability of LVEDP assessments. BNP and LVEDP were measured during a single study visit. While this limitation is inherent to most outpatient diagnostic assessments, it should be considered when interpreting the study findings. The cohort was clinically heterogeneous and includes many comorbidities that may affect BNP and LVEDP. Moreover, echocardiographic data were unavailable for left ventricular ejection fraction assessment, preventing adjustment for HF phenotype and introducing the potential for residual confounding. Lastly, to allow standardized assessment across all patients, LVEDP was determined using the R-peak of the ECG signal rather than the “Z” point at the end of the atrial (“A”) wave [32]. Since this approach can yield slightly higher values due to the rapid rise in pressure with the onset of ventricular contraction, the reference LVEDP values used in this study should be interpreted with this additional context.

## 6. Conclusions

BNP demonstrated greater diagnostic accuracy at higher LVEDP thresholds, but its limited sensitivity at lower thresholds may hinder the detection of hemodynamic abnormalities relevant to the evaluation of Stage B HF. Accordingly, BNP should be interpreted in the context of a broader clinical assessment and other diagnostic findings. Future studies should evaluate whether echocardiographic parameters, additional biomarkers, or other multimodal diagnostic strategies provide incremental value for identifying elevated filling pressure.

## Figures and Tables

**Table 1:** Patient characteristics and disease conditions for various LVEDP subgroups.

	Overall [154]	LVEDP Threshold: 15 mmHg		*p*-Value	LVEDP Threshold: 20 mmHg		*p*-Value	LVEDP Threshold: 25 mmHg		*p*-Value
		>Threshold [61, 40%]	≤Threshold [93, 60%]		>Threshold [32, 21%]	≤Threshold [122, 79%]		>Threshold [12, 8%]	≤Threshold [142, 92%]	
Patient Characteristics										
Age [years]	66 ± 10	66 ± 10	67 ± 10	0.412	65 ± 10	67 ± 10	0.573	65 ± 10	66 ± 10	0.720
BMI [kg/m^2^]	29 ± 5	30 ± 5	28 ± 5	**<0.001**	31 ± 4	28 ± 5	**0.011**	33 ± 5	28 ± 5	**0.006**
Male, *n* (%)	99 (64%)	38 (62%)	61 (66%)	0.806	19 (59%)	80 (66%)	0.657	6 (50%)	93 (65%)	0.446
Ethnicity, *n* (%)										
White	109 (71%)	48 (79%)	61 (66%)	0.117	27 (84%)	82 (67%)	0.093	7 (58%)	102 (72%)	0.511
Black	28 (18%)	8 (13%)	20 (22%)	0.268	4 (13%)	24 (20%)	0.497	4 (33%)	24 (17%)	0.304
Asian	14 (9%)	4 (7%)	10 (11%)	0.549	1 (3%)	13 (11%)	0.330	1 (8%)	13 (9%)	>0.999
Conditions, *n* (%)										
Hypertension	123 (80%)	49 (80%)	74 (80%)	>0.999	25 (78%)	98 (80%)	0.977	11 (92%)	112 (79%)	0.492
Diabetes	55 (36%)	25 (41%)	30 (32%)	0.351	13 (41%)	42 (34%)	0.657	7 (58%)	48 (34%)	0.165
Kidney Disease	21 (14%)	11 (18%)	10 (11%)	0.295	7 (22%)	14 (11%)	0.216	4 (33%)	17 (12%)	0.103
Lung Disease	71 (46%)	24 (39%)	47 (51%)	0.231	11 (34%)	60 (49%)	0.195	2 (17%)	69 (49%)	0.067
Liver Disease	3 (2%)	1 (2%)	2 (2%)	>0.999	1 (3%)	2 (2%)	>0.999	1 (8%)	2 (1%)	0.563
Heart Valve Disease	29 (19%)	12 (20%)	17 (18%)	>0.999	5 (16%)	24 (20%)	0.789	4 (33%)	25 (18%)	0.340
Coronary Artery Disease	45 (29%)	19 (31%)	26 (28%)	0.807	10 (31%)	35 (29%)	0.948	2 (17%)	43 (30%)	0.506
Atrial Fibrillation	19 (12%)	12 (20%)	7 (8%)	**0.046**	6 (19%)	13 (11%)	0.349	2 (17%)	17 (12%)	0.986
Heart Failure	37 (24%)	17 (28%)	20 (22%)	0.477	13 (41%)	24 (20%)	**0.025**	8 (67%)	29 (20%)	**0.001**
Lab Results										
LVEDP [mmHg]	15 ± 7	21 ± 6	10 ± 5	**<0.001**	25 ± 5	12 ± 5	**<0.001**	31 ± 4	13 ± 6	**<0.001**
BNP [pg/mL]	161 ± 367	234 ± 522	114 ± 202	**0.048**	360 ± 690	109 ± 188	**<0.001**	660 ± 1058	119 ± 190	**<0.001**

BMI: body mass index; LVEDP: left ventricular end-diastolic pressure; BNP: B-type natriuretic peptide. *p*-values < 0.05 are highlighted in bold.

**Table 2:** Patient characteristics and disease conditions for various BNP subgroups.

	Overall [154]	BNP Threshold: 35 pg/mL		*p*-Value	BNP Threshold: 100 pg/mL		*p*-Value
		>Threshold [93, 60%]	≤Threshold [61, 40%]		>Threshold [50, 32%]	≤Threshold [104, 68%]	
Patient Characteristics							
Age [years]	66 ± 10	68 ± 9	64 ± 9	**0.011**	69 ± 9	65 ± 10	**0.034**
BMI [kg/m^2^]	29 ± 5	28 ± 5	29 ± 5	0.396	29 ± 6	29 ± 5	0.709
Male, *n* (%)	99 (64%)	56 (60%)	43 (70%)	0.259	36 (72%)	63 (61%)	0.228
Ethnicity, *n* (%)							
White	109 (71%)	69 (74%)	40 (66%)	0.332	33 (66%)	76 (73%)	0.475
Black	28 (18%)	15 (16%)	13 (21%)	0.547	12 (24%)	16 (15%)	0.282
Asian	14 (9%)	7 (8%)	7 (11%)	0.584	4 (8%)	10 (10%)	0.978
Conditions, *n* (%)							
Hypertension	123 (80%)	74 (80%)	49 (80%)	>0.999	44 (88%)	79 (76%)	0.126
Diabetes	55 (36%)	40 (43%)	15 (25%)	**0.031**	26 (52%)	29 (28%)	**0.006**
Kidney Disease	21 (14%)	17 (18%)	4 (7%)	0.067	13 (26%)	8 (8%)	**0.004**
Lung Disease	71 (46%)	43 (46%)	28 (46%)	>0.999	23 (46%)	48 (46%)	>0.999
Liver Disease	3 (2%)	3 (3%)	0 (0%)	0.412	2 (4%)	1 (1%)	0.513
Heart Valve Disease	29 (19%)	24 (26%)	5 (8%)	**0.012**	14 (28%)	15 (14%)	0.072
Coronary Artery Disease	45 (29%)	32 (34%)	13 (21%)	0.117	19 (38%)	26 (25%)	0.141
Atrial Fibrillation	19 (12%)	15 (16%)	4 (7%)	0.130	11 (22%)	8 (8%)	**0.023**
Heart Failure	37 (24%)	29 (31%)	8 (13%)	**0.018**	25 (50%)	12 (12%)	**<0.001**
Lab Results							
LVEDP [mmHg]	15 ± 7	16 ± 9	13 ± 4	**0.028**	17 ± 10	14 ± 5	**0.006**
BNP [pg/mL]	161 ± 367	253 ±450	21 ± 9	**<0.001**	421 ± 564	37 ± 23	**<0.001**

BMI: body mass index; LVEDP: left ventricular end-diastolic pressure; BNP: B-type natriuretic peptide. *p*-values < 0.05 are highlighted in bold.

**Table 3:** Diagnostic metrics with 95% confidence intervals.

	LVEDP Threshold: 15 mmHg [61/154]		LVEDP Threshold: 20 mmHg [32/154]		LVEDP Threshold: 25 mmHg [12/154]	
AUC	0.65 (0.56, 0.74)		0.75 (0.65, 0.84)		0.81 (0.65, 0.97)	
BNP threshold	35 pg/mL	100 pg/mL	35 pg/mL	100 pg/mL	35 pg/mL	100 pg/mL
Sensitivity	0.74 (0.61, 0.84)	0.43 (0.30, 0.56)	0.88 (0.71, 0.96)	0.63 (0.44, 0.79)	0.92 (0.62, 1.00)	0.75 (0.43, 0.95)
Specificity	0.48 (0.38, 0.59)	0.74 (0.64, 0.83)	0.47 (0.38, 0.56)	0.75 (0.67, 0.83)	0.42 (0.34, 0.51)	0.71 (0.63, 0.78)
PPV	0.48 (0.38, 0.59)	0.52 (0.37, 0.66)	0.30 (0.21, 0.40)	0.40 (0.26, 0.55)	0.12 (0.06, 0.20)	0.18 (0.09, 0.31)
NPV	0.74 (0.61, 0.84)	0.66 (0.56, 0.75)	0.93 (0.84, 0.98)	0.88 (0.81, 0.94)	0.98 (0.91, 1.00)	0.97 (0.92, 0.99)
Positive Likelihood Ratio	1.43 (1.12, 1.83)	1.65 (1.05, 2.59)	1.64 (1.33, 2.03)	2.54 (1.69, 3.83)	1.59 (1.27, 1.98)	2.60 (1.71, 3.94)
Negative Likelihood Ratio	0.54 (0.34, 0.87)	0.77 (0.60, 0.99)	0.27 (0.10, 0.68)	0.50 (0.31, 0.79)	0.20 (0.03, 1.30)	0.35 (0.13, 0.94)

LVEDP: left ventricular end-diastolic pressure; BNP: B-type natriuretic peptide; AUC: area under the curve; PPV: positive predicative value; NPV: negative predictive value.

## Data Availability

Due to the sensitive and protected nature of the data collected for this study, the data are not publicly available and will remain under embargo for five years. Thereafter, deidentified data may be made available upon reasonable request to the corresponding author, subject to institutional approval and applicable data use agreements.

## Supplementary Materials

The following supporting information can be downloaded at https://www.mdpi.com/article/10.3390/diagnostics16162629/s1: Table S1: Multivariate analysis between potential confounders and BNP. Table S2: Diagnostic metrics with 95% confidence intervals after excluding patients with kidney disease diagnosis. Table S3: Diagnostic metrics with 95% confidence intervals after excluding patients with heart failure diagnosis.
