# Supplementary material for: Diagnostic Performance of Guideline-Recommended B-Type Natriuretic Peptide Thresholds for Detecting Elevated Left Ventricular End-Diastolic Pressure
Source: Diagnostics (Basel). 2026 Aug 19;16(16):2629. doi: 10.3390/diagnostics16162629 (PMC13512882; doi:10.3390/diagnostics16162629)
Supplement: Supplementary file 1 [file diagnostics-16-02629-s001.zip › diagnostics-4323727-supplementary.pdf]

Supplementary Materials

Table S1. Multivariate analysis between potential confounders and BNP.

	Coefficient	95% Confidence Interval	t-statistics	p-value
Constant	354.3	[-192.8, 901.4]	1.3	0.203
Age	-4.1	[-10.0, 1.8]	-1.4	0.168
BMI	-3.2	[-13.9, 7.6]	-0.6	0.560
Sex	38.5	[-78.0, 155.0]	0.7	0.515
Hypertension	74.9	[-65.4, 215.1]	1.1	0.293
Diabetes	62.3	[-56.6, 181.2]	1.0	0.302
Kidney Disease	216.3	[54.6, 378.1]	2.6	**0.009**
Lung Disease	-58.2	[-190.4, 74.0]	-0.9	0.386
Liver Disease	260.2	[-141.2, 661.5]	1.3	0.202
Heart Valve Disease	-17.7	[-163.6, 128.3]	-0.2	0.811
Coronary Artery Disease	-57.2	[-196.3, 81.9]	-0.8	0.417
Atrial Fibrillation	50.9	[-132.6, 234.3]	0.5	0.584
Heart Failure	294.1	[162.2, 425.9]	4.4	**< 0.001**

BMI: body mass index.

Table S2. Diagnostic metrics with 95% confidence intervals after excluding patients with kidney disease diagnosis.

	LVEDP threshold: 15 mmHg [50/133]		LVEDP threshold: 20 mmHg [25/133]		LVEDP threshold: 25 mmHg [8/133]	
AUC	0.62 (0.53, 0.72)		0.71 (0.60, 0.82)		0.74 (0.52, 0.96)	
BNP threshold	35 pg/mL	100 pg/mL	35 pg/mL	100 pg/mL	35 pg/mL	100 pg/mL
Sensitivity	0.70 (0.55, 0.82)	0.34 (0.21, 0.49)	0.84 (0.64, 0.95)	0.52 (0.31, 0.72)	0.88 (0.47, 1.00)	0.63 (0.24, 0.91)
Specificity	0.51 (0.39, 0.62)	0.76 (0.65, 0.85)	0.49 (0.39, 0.59)	0.78 (0.69, 0.85)	0.45 (0.36, 0.54)	0.74 (0.66, 0.82)
PPV	0.46 (0.35, 0.58)	0.46 (0.29, 0.63)	0.28 (0.18, 0.39)	0.35 (0.20, 0.53)	0.09 (0.04, 0.18)	0.14 (0.05, 0.29)
NPV	0.74 (0.60, 0.84)	0.66 (0.55, 0.75)	0.93 (0.83, 0.98)	0.88 (0.79, 0.93)	0.98 (0.91, 1.00)	0.97 (0.91, 0.99)
Positive Likelihood Ratio	1.42 (1.07, 1.88)	1.41 (0.82, 2.43)	1.65 (1.28, 2.12)	2.34 (1.40, 3.92)	1.59 (1.17, 2.15)	2.44 (1.32, 4.51)
Negative Likelihood Ratio	0.59 (0.37, 0.95)	0.87 (0.69, 1.10)	0.33 (0.13, 0.82)	0.62 (0.41, 0.94)	0.28 (0.04, 1.76)	0.50 (0.20, 1.24)

LVEDP: left ventricular end diastolic pressure; BNP: B-type natriuretic peptide; AUC: area under the curve; PPV: positive predicative value; NPV: negative predictive value.

Table S3. Diagnostic metrics with 95% confidence intervals after excluding patients with heart failure diagnosis.

	LVEDP threshold: 15 mmHg [44/117]		LVEDP threshold: 20 mmHg [19/117]		LVEDP threshold: 25 mmHg [4/117]	
AUC	0.62 (0.52, 0.73)		0.70 (0.56, 0.84)		0.60 (0.20, 1.00)	
BNP threshold	35 pg/mL	100 pg/mL	35 pg/mL	100 pg/mL	35 pg/mL	100 pg/mL
Sensitivity	0.66 (0.50, 0.80)	0.27 (0.15, 0.43)	0.79 (0.54, 0.94)	0.42 (0.20, 0.67)	0.75 (0.19, 0.99)	0.25 (0.01, 0.81)
Specificity	0.52 (0.40, 0.64)	0.82 (0.71, 0.90)	0.50 (0.40, 0.60)	0.83 (0.74, 0.90)	0.46 (0.37, 0.56)	0.79 (0.70, 0.86)
PPV	0.45 (0.33, 0.58)	0.48 (0.28, 0.69)	0.23 (0.14, 0.36)	0.32 (0.15, 0.54)	0.05 (0.01, 0.13)	0.04 (0.00, 0.20)
NPV	0.72 (0.58, 0.83)	0.65 (0.55, 0.75)	0.92 (0.82, 0.98)	0.88 (0.80, 0.94)	0.98 (0.90, 1.00)	0.97 (0.91, 0.99)
Positive Likelihood Ratio	1.37 (1.00, 1.89)	1.53 (0.77, 3.05)	1.58 (1.64, 2.14)	2.43 (1.23, 4.80)	1.39 (0.77, 2.51)	1.18 (0.21, 6.67)
Negative Likelihood Ratio	0.65 (0.41, 1.04)	0.88 (0.72, 1.09)	0.42 (0.17, 1.03)	0.70 (0.47, 1.04)	0.54 (0.09, 3.00)	0.95 (0.54, 1.69)

LVEDP: left ventricular end diastolic pressure; BNP: B-type natriuretic peptide; AUC: area under the curve; PPV: positive predicative value; NPV: negative predictive value.
